# Dimensional bone changes in ST1 and ST2 extraction sockets treated with an extracellular matrix: A CBCT-based observational pilot study

**DOI:** 10.4317/jced.64270

**Published:** 2026-09-15

**Authors:** Mario Guerrero-Torres, Maria Camila Olaciregui-Hernandez, Paola Andrea Lizarazo, Samir Salhem Eljadue-Machuca

**Affiliations:** 1 Facultad de Odontologia, Universidad Santo Tomas, Bucaramanga, Colombia

**Keywords:** Tooth extraction, tooth socket, extracellular matrix, cone-beam computed tomography, bone regeneration.

## Abstract

**Background:**

Evidence regarding dimensional changes in extracellular matrix (ECM)-treated extraction sockets stratified by buccal-wall morphology is limited. This study described 6-month CBCT changes in ST1 and ST2 sockets treated with porcine-derived ECM.

**Materials and Methods:**

This prospective observational pilot case series included nine sockets (five ST1 and four ST2) treated with microlaminated porcine-derived ECM and an ECM membrane. Pre-extraction, 3-month, and 6-month CBCT volumes were rigidly registered to reproduce the measurement site. A single experienced examiner recorded vertical and cervical, middle, and apical widths. A randomly selected measurement subset was repeated after approximately 3 months to assess intra-examiner reliability. Outcomes were summarized descriptively as mean ± SD.

**Results:**

The intra-examiner ICC was 0.88, indicating good reliability. At 6 months, ST1 sockets showed mean changes of -0.40 ± 0.96 mm vertically, -1.46 ± 1.52 mm cervically, -0.64 ± 1.62 mm at the middle level, and +1.24 ± 1.91 mm apically. ST2 sockets showed changes of -0.07 ± 0.82 mm, -2.45 ± 0.50 mm, -2.30 ± 1.10 mm, and -1.45 ± 0.52 mm, respectively. One site reported early postoperative symptoms managed with analgesics; no infection, wound instability, delayed healing, or loss to follow-up occurred.

**Conclusions:**

The ECM protocol was clinically feasible and associated with small mean vertical changes, while horizontal remodeling varied with baseline socket morphology. ST1 sockets showed smaller cervical and middle reductions than ST2, supporting controlled evaluation of ECM across contained and fenestrated sockets.

## Introduction

Tooth extraction initiates alveolar remodeling that reduces ridge dimensions and may complicate implant positioning and prosthetic rehabilitation. Horizontal contraction is generally greater than vertical change, particularly at the buccal-coronal aspect, where the facial plate is frequently thin and largely composed of bundle bone [1-4]. Prospective evidence also indicates that sites with thin facial bone undergo greater linear and volumetric reduction than sites with thicker facial bone [5].

Socket morphology therefore represents an important determinant of post-extraction dimensional change. In the classification proposed by Steigmann *et al*. [6], ST1 sockets retain an intact buccal wall and are subdivided into ST1A (buccal wall thickness ≥1 mm) and ST1B (<1 mm). ST2 sockets present a buccal-wall fenestration without loss of crestal height, whereas defects with crestal dehiscence constitute a different and more complex category. Distinguishing intact from fenestrated sockets may help interpret dimensional changes after ridge-preservation procedures.

Alveolar ridge preservation can attenuate, but does not eliminate, post-extraction remodeling [7]. Protocols often combine an intra-socket material with a socket-covering barrier to stabilize the healing compartment [8]. Extracellular matrix (ECM) biomaterials provide collagen-rich biological scaffolds that may support cell adhesion, migration, angiogenesis, and tissue organization [9,10]. Nevertheless, clinical evidence on ECM use in extraction sockets stratified by buccal-wall morphology remains limited.

CBCT permits longitudinal assessment of ridge dimensions, although radiographic stability cannot be equated with histologically confirmed vital bone [11]. Accordingly, the objective of this prospective observational pilot study was to describe vertical and horizontal dimensional changes in ST1 and ST2 extraction sockets treated with porcine-derived ECM over 6 months and to document the postoperative clinical course of the treated sites.

## Materials and Methods

*Study design*

This was a prospective, longitudinal, observational pilot case series. Participants were enrolled before extraction and followed clinically and radiographically for 6 months. The study was conducted from June 2025 to April 2026 at the Periodontics and Osseointegration Clinics of Universidad Santo Tomas, located at the Centro Internacional de Especialistas in the Bucaramanga metropolitan area, Colombia. Applicable items of the STROBE Statement were used to structure the report [12].

The protocol was approved by the institutional Research Ethics Committee of Universidad Santo Tomas (approval No. 0263-2025; April 24, 2025). All participants provided written informed consent before enrollment and authorized the use of non-identifiable clinical and tomographic information for research and publication.

*Participants recruitment*

Recruitment used non-probability convenience sampling. Potential candidates were identified among patients attending the university Periodontics and Osseointegration Clinics and through referral by clinic personnel; recruitment was therefore not consecutive. A clinic-wide screening log was not maintained. The pilot sample comprised participants who met the clinical and socket-morphology criteria and completed all three planned CBCT examinations during the study period. The study was designed to apply a standardized surgical and serial-CBCT workflow and generate preliminary descriptive estimates; therefore, no hypothesis-based sample-size calculation was performed.

Adults were eligible when they were systemically healthy or medically controlled, required extraction of a non-restorable anterior or posterior tooth followed by ridge preservation, had an ST1 or ST2 socket without an apical lesion at the treated site, accepted participation and the planned CBCT schedule, and had an interpretable baseline CBCT examination. Exclusion criteria were inability to provide informed consent, pregnancy, uncontrolled systemic disease, active periodontal disease, socket morphologies other than ST1 or ST2, previous maxillofacial radiotherapy, uncontrolled coagulation disorders, active oncologic treatment or clinically significant immunosuppression, medication-related high risk of osteonecrosis of the jaws, or imaging artifacts that prevented reproducible measurement. The socket was the unit of radiomorphometric analysis; some participants contributed more than one socket.

*Socket classification*

Socket morphology was classified according to the condition of the buccal plate. ST1 indicated an intact buccal wall; ST1A denoted a wall thickness ≥1 mm and ST1B a thickness <1 mm. ST2 indicated a fenestration of the buccal wall without loss of crestal height. Classification was based on preoperative clinical and CBCT assessment and was confirmed by direct inspection after extraction. ST1A and ST1B were combined for analysis.

*Surgical protocol*

The porcine-derived xenogeneic ECM (ActivaMec) was used as a microlaminated intra-socket filling material and as a membrane covering the socket entrance. Local anesthesia was achieved with 2% lidocaine containing epinephrine 1:80,000. All procedures were performed by final-year postgraduate periodontics residents under a standardized protocol.

Patients rinsed with 0.12% chlorhexidine for 60 seconds. Extraction was performed atraumatically and without systematic flap elevation. Circumferential supracrestal and periodontal ligament fibers were severed with a fine periotome, followed by controlled luxation. Odontosection was permitted when required by root anatomy. Ostectomy and intentional reduction of the socket walls were not performed as part of the protocol. After extraction, the socket was curetted and irrigated with sterile saline.

Hydrated microlaminated ECM was placed within the socket. For ST1 sockets, the ECM membrane was adapted over the entrance. For ST2 sockets, the membrane was trimmed into a V-shaped configuration and extended toward the buccal fenestration before the socket was filled, following an ice-cream-cone containment approach. The membrane was positioned without pressure and stabilized with 5-0 nylon sutures without forced primary closure.

*Postoperative care and follow-up*

Naproxen 500 mg was prescribed every 8 hours for 3 days. Systemic antibiotics were not included in the standardized postoperative regimen. Participants received verbal and written instructions and were assessed by telephone at 24 hours and clinically at 8 days, 15 days, 3 months, and 6 months. Pain, edema, bleeding, infection, wound instability, delayed healing, biomaterial exposure, and any condition requiring additional treatment were recorded.

*CBCT acquisition, registration, and measurements*

CBCT examinations were obtained at three time points: A pre-extraction scan used as the baseline (T0) and postoperative scans at 3 months (T1) and 6 months (T2). All examinations were acquired with the same Dentsply Sirona CBCT unit under equivalent exposure settings within each participant. DICOM files were imported into BlueSkyPlan version 5.1. At T0, the tooth was digitally removed during segmentation so that the socket region could be characterized from the pre-extraction volume.

The T1 and T2 volumes were rigidly registered to T0 using stable maxillary structures outside the remodeling site. Registration was verified in axial, coronal, and sagittal planes. A cross-sectional slice through the geometric center of the socket and perpendicular to the alveolar curvature was selected at T0; its orientation and location were then transferred to T1 and T2.

Four linear dimensions were recorded on the standardized cross-section. The vertical dimension was the distance from the most coronal crestal point at the treated site to the apex of the adjacent mesial tooth; when the mesial tooth was absent, the distal tooth apex was used. The same dental reference was retained at all time points. Horizontal dimensions represented the total buccopalatal or buccolingual width between the external cortical limits. The cervical level corresponded to the coronal limit established on the baseline socket, the middle level to the midpoint of the baseline vertical reference, and the apical level to the apical limit established at T0. Lines were drawn perpendicular to the socket longitudinal axis, and the baseline levels were transferred unchanged to the registered follow-up volumes.

All radiomorphometric measurements were performed by a single trained examiner with more than 10 years of experience in CBCT analysis. The same examiner evaluated all scans using BlueSkyPlan, the registered cross-sectional plane, standardized anatomical landmarks, and predefined measurement criteria across all time points. To assess intra-examiner repeatability, a randomly selected subset of CBCT measurements was repeated by the same examiner after an approximately 3-month interval, under the same software and anatomical criteria and without consulting the initial values.

*Outcomes and statistical analysis*

Primary outcomes were absolute vertical, cervical, middle, and apical dimensional changes from T0 to T1 and T2. Change was calculated as follow-up minus baseline; positive values indicated gain and negative values indicated reduction. Relative change was calculated as [(follow-up - baseline)/baseline] x 100. Secondary outcomes were clinical healing and postoperative events.

Data were entered in Microsoft Excel and analyzed in STATA version 14.0. Quantitative measurements were summarized using socket-level values and mean ± sample standard deviation (SD) by socket category. Categorical outcomes were reported as counts. Intra-examiner repeatability was estimated using a two-way mixed-effects, absolute-agreement, single-measure intraclass correlation coefficient (ICC). ICC values <0.50, 0.50-0.75, 0.75-0.90, and >0.90 were interpreted as poor, moderate, good, and excellent reliability, respectively [13]. No hypothesis testing, adjusted modeling, imputation, or confirmatory comparison between ST1 and ST2 was performed. Participant-level clustering from multiple sockets was not modeled; all group comparisons are descriptive.

## Results

Six participants contributed nine treated sockets with complete baseline, 3-month, and 6-month measurements. Five sockets were classified as ST1 and four as ST2. At the socket level, eight sites corresponded to female participants and one to a male participant; recorded ages ranged from 37 to 54 years. Baseline characteristics are shown in Table 1. Repeated assessment of the randomly selected measurement subset yielded an ICC of 0.88, indicating good intra-examiner reliability according to the predefined criteria.

All sockets were treated according to the planned ECM protocol. One ST1 site (Site 9; tooth 27) reported early postoperative symptoms at the 24-hour assessment, which were managed with the prescribed analgesic regimen. Membrane resorption was clinically evident at 8 days; the membrane was no longer visible at 15 days in Sites 5 and 9. No infection, wound instability, delayed healing, or event that compromised the planned follow-up was recorded. The clinical sequence is summarized in Figure 1.

Complete socket-level measurements and 6-month changes are provided in Tables 2 and 3. Group summaries with mean ± SD are presented in Table 4. The registration and measurement workflow is shown in Figure 2, and the sagittal CBCT sequence is shown in Figure 3.

Between baseline and 3 months, mean vertical change was small in both categories (-0.30 mm in ST1 and -0.12 mm in ST2), while horizontal changes were more evident. From 3 to 6 months, mean cervical width decreased by a further 0.38 mm in ST1 and 1.09 mm in ST2; middle width changed by +0.14 mm and -0.53 mm, respectively. During the same interval, apical width changed by +0.16 mm in ST1 and -0.35 mm in ST2.

At 6 months, ST1 sockets had mean changes of -0.40 ± 0.96 mm vertically, -1.46 ± 1.52 mm cervically, -0.64 ± 1.62 mm at the middle level, and +1.24 ± 1.91 mm apically. The corresponding group-relative changes were -3.1%, -17.5%, -6.6%, and +12.1%, respectively.

ST2 sockets had mean 6-month changes of -0.07 ± 0.82 mm vertically, -2.45 ± 0.50 mm cervically, -2.30 ± 1.10 mm at the middle level, and -1.45 ± 0.52 mm apically. The corresponding group-relative changes were -0.4%, -31.3%, -27.1%, and -17.4%. Thus, vertical mean change was small in both categories, whereas mean cervical and middle reductions were larger in ST2 sockets. These data are descriptive and do not establish a between-group effect.

## Discussion

This prospective observational pilot case series described 6-month clinical and radiomorphometric changes in ST1 and ST2 sockets treated with porcine-derived ECM. Three findings merit emphasis: The mean vertical dimension changed little in either socket category; horizontal remodeling was more pronounced and differed by baseline buccal-wall morphology; and the selected sites followed a generally uncomplicated clinical course. ST1 sockets showed smaller mean cervical and middle reductions and a mean apical increase, whereas ST2 sockets showed reductions at all three horizontal levels.

The predominance of horizontal over vertical reduction agrees with established post-extraction remodeling patterns. Couso-Queiruga *et al*. [3] reported pooled radiographic horizontal reductions of approximately 2.54 mm in non-molar and 3.61 mm in molar sites after unassisted healing, with smaller vertical changes. In the present series, mean cervical reduction was 1.46 mm in ST1 and 2.45 mm in ST2 sockets. These figures should be compared cautiously because the present baseline was the pre-extraction CBCT, whereas many studies use an immediate post-extraction or post-treatment scan. The direction of change nevertheless aligns with evidence that facial bone morphology influences subsequent linear and volumetric remodeling [4,5].

The protocol combined microlaminated ECM within the socket with an ECM membrane at the socket entrance. Systematic and controlled evidence indicates that ridge-preservation and socket-sealing procedures can attenuate, but do not eliminate, post-extraction dimensional change [7,8,14,15]. Against that background, the absence of infection, wound instability, delayed healing, or follow-up loss supports the clinical feasibility of the ECM-based workflow in the selected ST1 and ST2 sites. Because this study had no untreated or active comparator, the magnitude of dimensional preservation attributable specifically to the ECM cannot be isolated.

The measurement workflow also addressed an important source of uncertainty in longitudinal CBCT studies. Rigid registration and transfer of the baseline cross-section reproduced the region of interest, while one examiner with more than 10 years of CBCT-analysis experience applied the same anatomical criteria at all time points. The repeated-measurement ICC of 0.88 represents good intra-examiner reliability under the prespecified interpretation [13]. This value is consistent with the range reported for standardized post-extraction CBCT measurements and supports the repeatability of the present protocol, while recognizing that an ICC point estimate does not by itself quantify systematic bias or the absolute error surrounding each millimetric measurement [16].

The observed patterns should also be interpreted in relation to the biological and physical roles of the material. ECM biomaterials are collagen-rich, remodelable scaffolds intended to support cell attachment, migration, angiogenesis, and tissue organization [9,10]. At the same time, stable space maintenance is a central requirement for bone regeneration, particularly when osseous containment is incomplete [17]. Clinical studies of ECM or collagen matrices used as socket barriers show that they can support healing and socket sealing, but continued hard-tissue remodeling is common and hard-tissue outcomes depend on the complete graft-barrier construct rather than on the seal alone [18,19]. Thus, the present data are compatible with a biologically supportive role for ECM but do not demonstrate superiority over particulate grafts, other membranes, or spontaneous healing.

In ST2 sites, the membrane was extended toward the buccal fenestration using an ice-cream-cone containment approach. Although no wound instability or delayed healing was documented, the larger descriptive cervical and middle reductions suggest that a flexible ECM construct may provide less resistance to facial soft-tissue pressure when the buccal plate is incomplete. This is a plausible mechanical interpretation rather than a tested causal mechanism. The contrast between ST1 and ST2 reinforces the clinical relevance of recording baseline socket morphology and suggests that contained and non-contained defects should not be pooled indiscriminately in future ridge-preservation studies [5,6,17].

The mean apical increase in ST1 sockets is an additional radiomorphometric observation of interest. In a contained socket, clot stabilization and vascular access from the apical and lateral walls could favor organization of the healing compartment; however, the present imaging cannot identify the tissue responsible for the increase. CBCT depicts mineralized radiopacity and may include newly formed bone, remodeled native bone, and residual mineralized components. Correlative histology is therefore required before the apical finding can be interpreted as regeneration [11].

From a clinical perspective, the smaller mean cervical and middle reductions in ST1 sockets correspond to greater retained horizontal dimension than in ST2 sockets within this sample and may be relevant to prosthetically driven implant planning. The larger ST2 reductions indicate a need for careful three-dimensional reassessment before implant surgery. Evidence from implant-treatment cohorts shows that ridge preservation and greater baseline facial bone thickness are associated with a lower need for ancillary augmentation [20]. The current study did not include implant-placement, re-entry, or augmentation outcomes, so it cannot determine whether the measured differences altered treatment complexity; it does, however, provide morphology-specific estimates that can inform those endpoints in subsequent trials.

The study was intentionally exploratory and included six participants and nine sockets recruited by convenience sampling, without an untreated or active comparator; some participants contributed more than one socket. The pre-extraction baseline, combined analysis of ST1A and ST1B, 6-month follow-up, linear rather than volumetric analysis, and absence of histology and patient-reported outcomes define the scope of interpretation. Within that scope, the prospective design, standardized surgical protocol, complete serial follow-up, rigid image registration, availability of socket-level data, and demonstrated intra-examiner repeatability strengthen the descriptive contribution of the study.

These observations provide a practical foundation for confirmatory research. Future studies should use documented consecutive screening and an a priori sample-size calculation; compare ECM with unassisted healing and an active ridge-preservation protocol; stratify or randomize by ST1/ST2 morphology and model patient-level clustering; and combine linear measurements with three-dimensional surface or volumetric analyses. Histomorphometry at implant-site re-entry could clarify the composition of the apical radiographic increase, while implant feasibility, need for ancillary augmentation, patient-reported outcomes, and longer-term peri-implant tissue stability would establish whether the radiomorphometric patterns translate into meaningful clinical benefit [20].

## Conclusions

Within the conditions of this prospective observational pilot study, ECM-treated ST1 and ST2 extraction sockets showed a generally uncomplicated clinical course and small mean vertical changes through 6 months. Horizontal remodeling remained morphology dependent: ST1 sockets showed smaller mean cervical and middle reductions together with a mean apical increase, whereas ST2 sockets showed larger mean reductions across the cervical, middle, and apical levels.

These observations support the feasibility and potential clinical value of the ECM protocol as a biologically oriented preservation approach, particularly in contained ST1 sockets. The more pronounced ST2 pattern suggests that buccal-wall fenestration may require additional space-maintaining strategies. Controlled, adequately powered studies stratified by socket morphology are needed to determine comparative efficacy and whether these radiographic patterns reduce the need for augmentation at implant placement.

## Figures and Tables

**Table 1:** Table 1: Baseline characteristics and treated sites.

**Socket category**	**Site code**	**Tooth/site (FDI)**	**Anatomical site**	**Baseline vertical (mm)**	**Baseline cervical (mm)**	**Baseline middle (mm)**	**Baseline apical (mm)**
ST1	Site 1 (F/47)	22	Left maxillary lateral incisor	15.5	6.1	7.3	9.5
ST1	Site 4 (F/54)	15	Right maxillary second premolar	12.7	5.2	7.1	7.6
ST1	Site 5 (M/37)	15	Right maxillary second premolar	13.6	7.4	9.1	11.7
ST1	Site 8 (F/40)	15	Right maxillary second premolar	10.8	10.3	10.7	11.8
ST1	Site 9 (F/47)	27	Left maxillary second molar	11.3	12.8	14.2	10.8
ST2	Site 2 (F/54)	13	Right maxillary canine	17.3	7.0	7.4	7.0
ST2	Site 3 (F/54)	14	Right maxillary first premolar	19.8	6.6	7.7	7.6
ST2	Site 6 (F/48)	11	Right maxillary central incisor	22.4	8.0	9.1	7.6
ST2	Site 7 (F/40)	25	Left maxillary second premolar	11.0	9.7	9.7	11.2

FDI: Federation Dentaire Internationale tooth numbering system.

**Table 2:** Table 2: Complete socket-level measurements at pre-extraction baseline, 3 months, and 6 months.

**Socket category**	**Site code**	**Time point**	**Vertical (mm)**	**Cervical (mm)**	**Middle (mm)**	**Apical (mm)**
ST1	Site 1 (F/47)	Pre-extraction baseline	15.5	6.1	7.3	9.5
ST1	Site 1 (F/47)	3 months	16.7	5.4	7.6	9.9
ST1	Site 1 (F/47)	6 months	16.2	4.8	8.8	10.1
ST1	Site 4 (F/54)	Pre-extraction baseline	12.7	5.2	7.1	7.6
ST1	Site 4 (F/54)	3 months	12.7	3.9	5.2	6.3
ST1	Site 4 (F/54)	6 months	13.1	3.7	5.0	6.5
ST1	Site 5 (M/37)	Pre-extraction baseline	13.6	7.4	9.1	11.7
ST1	Site 5 (M/37)	3 months	11.6	7.7	9.3	14.6
ST1	Site 5 (M/37)	6 months	12.3	8.0	9.8	15.1
ST1	Site 8 (F/40)	Pre-extraction baseline	10.8	10.3	10.7	11.8
ST1	Site 8 (F/40)	3 months	10.2	8.2	9.9	11.6
ST1	Site 8 (F/40)	6 months	9.4	6.6	9.0	12.1
ST1	Site 9 (F/47)	Pre-extraction baseline	11.3	12.8	14.2	10.8
ST1	Site 9 (F/47)	3 months	11.2	11.2	12.5	14.4
ST1	Site 9 (F/47)	6 months	10.9	11.4	12.6	13.8
ST2	Site 2 (F/54)	Pre-extraction baseline	17.3	7.0	7.4	7.0
ST2	Site 2 (F/54)	3 months	19.1	5.2	5.7	6.8
ST2	Site 2 (F/54)	6 months	17.6	4.0	5.1	5.6
ST2	Site 3 (F/54)	Pre-extraction baseline	19.8	6.6	7.7	7.6
ST2	Site 3 (F/54)	3 months	20.7	5.4	5.8	6.2
ST2	Site 3 (F/54)	6 months	18.6	4.0	5.3	6.5
ST2	Site 6 (F/48)	Pre-extraction baseline	22.4	8.0	9.1	7.6
ST2	Site 6 (F/48)	3 months	21.8	6.7	6.5	5.7
ST2	Site 6 (F/48)	6 months	22.3	6.2	5.5	5.4
ST2	Site 7 (F/40)	Pre-extraction baseline	11.0	9.7	9.7	11.2
ST2	Site 7 (F/40)	3 months	8.4	8.6	8.8	10.3
ST2	Site 7 (F/40)	6 months	11.7	7.3	8.8	10.1

All values are expressed in millimeters. The pre-extraction baseline was used as the reference for longitudinal comparisons.

**Table 3:** Table 3: Six-month socket-level dimensional change relative to pre-extraction baseline.

**Category**	**Site code**	**Tooth**	**Vertical change**	**Cervical change**	**Middle change**	**Apical change**
ST1	Site 1 (F/47)	22	+0.7 mm (+4.5%)	-1.3 mm (-21.3%)	+1.5 mm (+20.5%)	+0.6 mm (+6.3%)
ST1	Site 4 (F/54)	15	+0.4 mm (+3.1%)	-1.5 mm (-28.8%)	-2.1 mm (-29.6%)	-1.1 mm (-14.5%)
ST1	Site 5 (M/37)	15	-1.3 mm (-9.6%)	+0.6 mm (+8.1%)	+0.7 mm (+7.7%)	+3.4 mm (+29.1%)
ST1	Site 8 (F/40)	15	-1.4 mm (-13.0%)	-3.7 mm (-35.9%)	-1.7 mm (-15.9%)	+0.3 mm (+2.5%)
ST1	Site 9 (F/47)	27	-0.4 mm (-3.5%)	-1.4 mm (-10.9%)	-1.6 mm (-11.3%)	+3.0 mm (+27.8%)
ST2	Site 2 (F/54)	13	+0.3 mm (+1.7%)	-3.0 mm (-42.9%)	-2.3 mm (-31.1%)	-1.4 mm (-20.0%)
ST2	Site 3 (F/54)	14	-1.2 mm (-6.1%)	-2.6 mm (-39.4%)	-2.4 mm (-31.2%)	-1.1 mm (-14.5%)
ST2	Site 6 (F/48)	11	-0.1 mm (-0.4%)	-1.8 mm (-22.5%)	-3.6 mm (-39.6%)	-2.2 mm (-28.9%)
ST2	Site 7 (F/40)	25	+0.7 mm (+6.4%)	-2.4 mm (-24.7%)	-0.9 mm (-9.3%)	-1.1 mm (-9.8%)

Positive values indicate dimensional gain relative to the pre-extraction baseline; negative values indicate dimensional reduction.

**Table 4:** Table 4: Descriptive dimensional outcomes by socket category (mean ± SD).

**Socket category**	**Measurement level**	**Baseline (mm)**	**3 months (mm)**	**6 months (mm)**	**Mean 6-month change (mm; group-relative %)**
ST1	Vertical	12.78 ± 1.88	12.48 ± 2.52	12.38 ± 2.56	-0.40 ± 0.96 (-3.1%)
ST1	Cervical	8.36 ± 3.14	7.28 ± 2.80	6.90 ± 3.01	-1.46 ± 1.52 (-17.5%)
ST1	Middle	9.68 ± 2.92	8.90 ± 2.72	9.04 ± 2.72	-0.64 ± 1.62 (-6.6%)
ST1	Apical	10.28 ± 1.76	11.36 ± 3.45	11.52 ± 3.38	+1.24 ± 1.91 (+12.1%)
ST2	Vertical	17.62 ± 4.88	17.50 ± 6.17	17.55 ± 4.39	-0.07 ± 0.82 (-0.4%)
ST2	Cervical	7.83 ± 1.38	6.47 ± 1.56	5.38 ± 1.65	-2.45 ± 0.50 (-31.3%)
ST2	Middle	8.48 ± 1.10	6.70 ± 1.44	6.17 ± 1.76	-2.30 ± 1.10 (-27.1%)
ST2	Apical	8.35 ± 1.92	7.25 ± 2.08	6.90 ± 2.19	-1.45 ± 0.52 (-17.4%)

Values are mean ± sample standard deviation. Group-relative percentage was calculated from the unrounded group means. No inferential comparison was performed.

**Figure 1:**
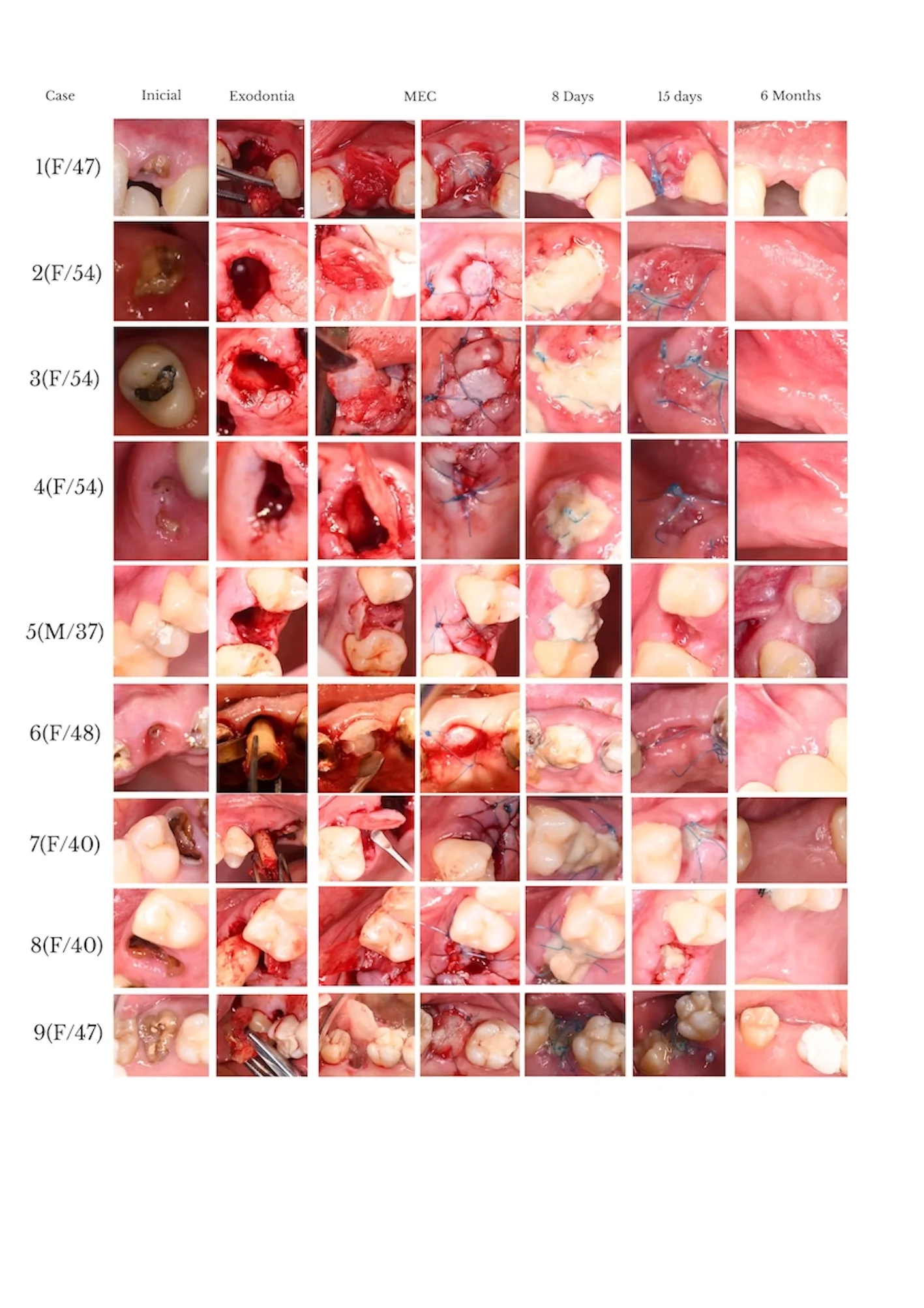
Fig. 1: Clinical photographic sequence of the treated extraction sockets. Intraoral photographs show the initial clinical condition, extraction, extracellular matrix placement, and healing at 8 days, 15 days, and 6 months. Sites are identified according to gender and age codes: F, female; M, male.

**Figure 2:**
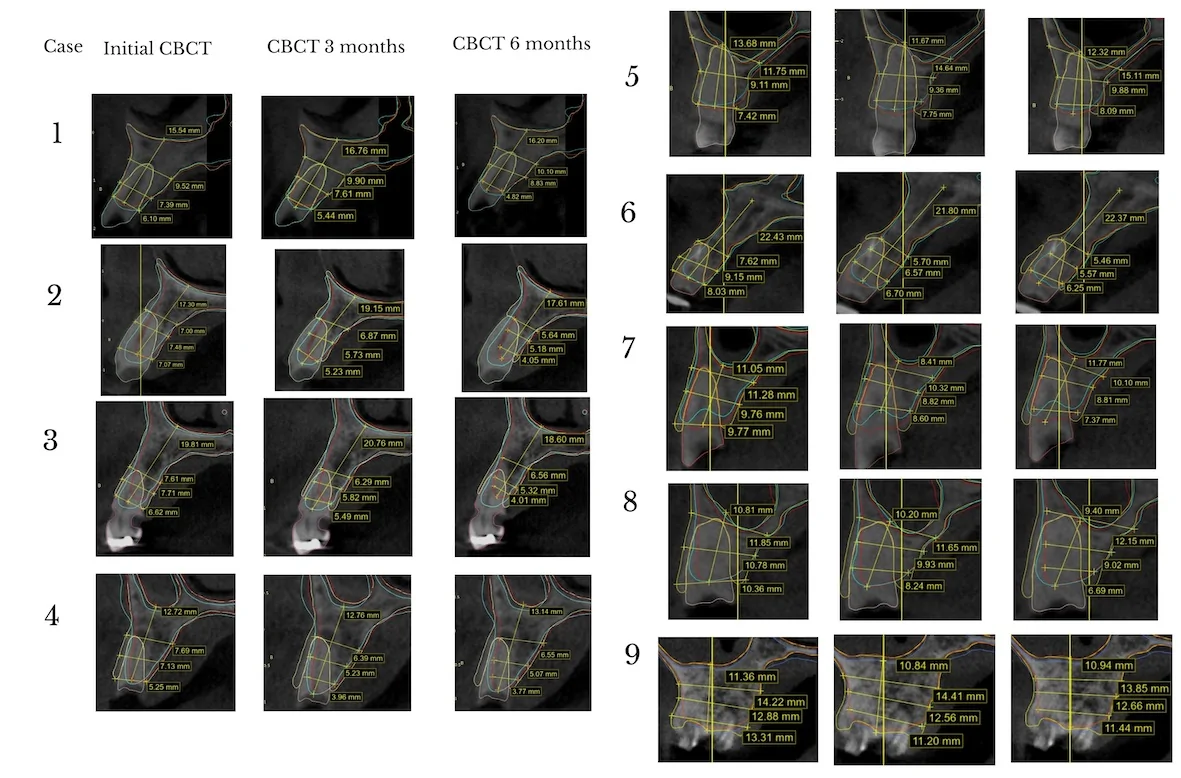
Fig. 2: CBCT registration and radiomorphometric measurements. Registered CBCT reconstructions show the protocol applied at the pre-extraction baseline, 3 months, and 6 months. One vertical measurement and three horizontal measurements at cervical, middle, and apical levels were obtained at the same transferred baseline location. Values are expressed in millimeters.

**Figure 3:**
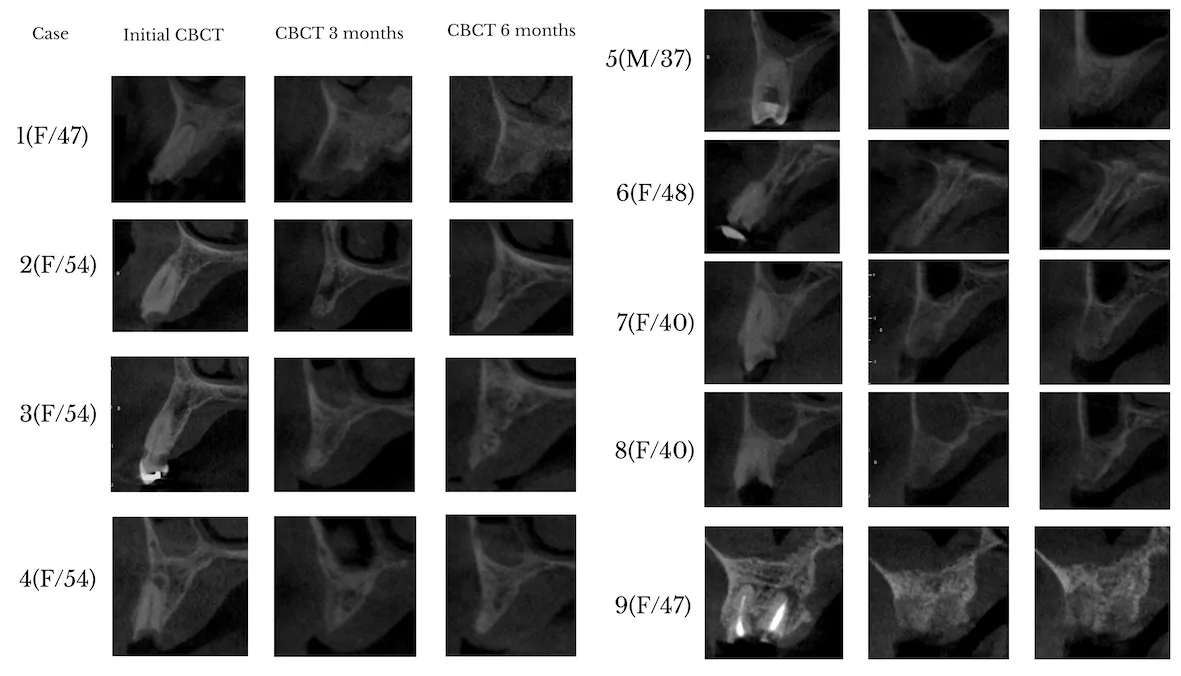
Fig. 3: Sagittal CBCT follow-up of the treated extraction sockets. Sagittal sections show the treated sites at the pre-extraction baseline, 3 months, and 6 months and provide a qualitative overview before quantitative radiomorphometric assessment.

## Data Availability

Declared none.
